# Western scrub-jays (*Aphelocoma californica*) solve multiple-string problems by the spatial relation of string and reward

**DOI:** 10.1007/s10071-016-1018-x

**Published:** 2016-07-28

**Authors:** M. M. Hofmann, L. G. Cheke, N. S. Clayton

**Affiliations:** 1 Department of Psychology, University of Cambridge, Downing Site, Cambridge, CB2 3EB UK; 2 Department of Biology, Ludwig-Maximilians-University Munich, Großhaderner Str. 2, 82152 Planegg-Martinsried, Germany

**Keywords:** String-pulling, Physical cognition, Corvids, Problem-solving, Causal reasoning

## Abstract

String-pulling is a widely used paradigm in animal cognition research to assess what animals understand about the functionality of strings as a means to obtain an out-of-reach reward. This study aimed to systematically investigate what rules Western scrub-jays (*Aphelocoma californica*) use to solve different patterned string tasks, i.e. tasks in which subjects have to choose between two or more strings of which only one is connected to the reward, or where one is more efficient. Arranging strings in a parallel configuration showed that the jays were generally capable of solving multiple-string tasks and acted in a goal-directed manner. The slanted and crossed configurations revealed a reliance on a “proximity rule”, that is, a tendency to choose the string-end closest to the reward. When confronted with strings of different lengths attached to rewards at different distances the birds chose according to the reward distance, preferring the reward closest to them, and were sensitive to the movement of the reward, but did not consistently prefer the shorter and therefore more efficient string. Generally, the scrub-jays were successful in tasks where the reward was closest to the string-ends they needed to pull or when string length and reward distance correlated, but the birds had problems when the wrong string-end was closest to the reward or when the food items were in close proximity to each other. These results show that scrub-jays had a partial understanding of the physical principles underlying string-pulling but relied on simpler strategies such as the proximity rule to solve the tasks.

**Electronic supplementary material:**

The online version of this article (doi:10.1007/s10071-016-1018-x) contains supplementary material, which is available to authorized users.

## Introduction

Corvids are known for their “exceptional memory, enormous curiosity, attractive movements, high sociability, varied vocalizations, and ecological plasticity” (Del Hoyo et al. 1992 vol 14, p. 494). It is therefore unsurprising that they are one of the key targets for the exploration of animal intelligence and have been referred to as “feathered apes” for their cognitive abilities (Emery 2004; Emery and Clayton 2004). It has been argued that corvids are especially skilled in the field of physical cognition, with reports of at least 24 tool-using species of corvids (Lefebvre et al. 2002).

The majority of research on physical cognition has been conducted with the New Caledonian crow (*Corvus moneduloides*). These birds are prolific tool users in the wild (Hunt 2014) and have demonstrated impressive physical cognition abilities in the laboratory (Chappell and Kacelnik 2002, 2004; Jelbert et al. 2014; Taylor et al. 2011; Weir et al. 2002). However, it appears that it is not necessary for a bird to be a tool-user in the wild in order to demonstrate tool-use in captivity. Rooks (*Corvus frugilegus*) have been shown to be able to choose functional tools or creatively modify nonfunctional ones to retrieve a reward (Bird and Emery 2009a), and both rooks and Eurasian jays can use stones as tools to raise the level of water (Bird and Emery 2009b; Cheke et al. 2011). Thus, it seems that corvids may possess a flexible cognitive “tool kit” that allows them to solve novel tasks even if they do not face similar challenges in their natural environment (Emery and Clayton 2004).

One famous paradigm with which to study physical cognition is string-pulling. In these experiments, a food item is placed within an animal’s field of vision but out of its reach. The reward is attached to a string, the end of which can be accessed by the animal, who may then obtain the food item by pulling the string. The strings can be arranged either horizontally (e.g. the food must be pulled under/through a barrier) or vertically (e.g. the food must be pulled up to a perch or platform). String-pulling tasks have been employed to test the motor skills and cognitive abilities of a variety of species. The complexity of these tasks can be manipulated by varying the number and alignment of strings, allowing the investigation of different aspects of cognition. For example, so-called patterned string problems (i.e. tasks with more than one string arranged in different patterns) confront subjects with slanted, crossed or otherwise misleading strings, of which only one is connected to the food, or one is more efficient in obtaining the food (e.g. Dücker and Rensch 1977; Schuck-Paim et al. 2008; Werdenich and Huber 2006). These tasks allow investigation of whether or not the animals take account of the causal association between the string and the food, or make their responses according to simpler rules such as “always choose the string-end which is closest to the reward” (the “proximity rule”). Exhaustive reviews of the existing string-pulling literature were made by Wasserman et al. (2013) and Jacobs and Osvath (2015).

Studies conducted within the genus *Corvus* indicate that these birds are capable of performance in string-pulling tasks comparable to that of monkeys and apes. Heinrich (1995) found that some, but not all, ravens (*C. corax*) tested could solve tasks with crossed or slanted strings, suggesting that they had an appreciation of the need for connectedness for the string to be a useful tool. Bagotskaya et al. (2012) demonstrated that hooded crows (*C. cornix*) are also capable of solving slanted-string tasks, but struggle with crossed strings. Manipulation of task design can reveal the limitations of corvid understanding of string problem. For example, introducing a visual restriction and thus preventing perceptual feedback leads to weak performances by both experienced and naive New Caledonian crows in otherwise well-solved string tasks in both horizontal and vertical set-ups (Taylor et al. 2010, 2012). Crows with no experience in string-pulling were not able to solve a vertical string task when visual feedback was restricted and even experienced birds did worse than when visual feedback was available. With one exception, naive wild crows were also not able to succeed in gaining a reward if they did not get visual feedback of the approaching food when distinguishing between connected and unconnected strings in a horizontal configuration. When the movement of the reward was prevented by the slack in the string, the birds stopped interacting with the apparatus. This result suggests that the birds rely on the approach of the food as a reinforcer for their actions. Based on these findings Taylor and colleagues conclude that it is not planning and complex cognition that underlie successful string-pulling, but a “perceptual-motor feedback cycle” (Taylor et al. 2010, p. 1).

The debate about the cognition behind string-pulling is ongoing. It remains unclear whether and to what degree cognitive understanding contributes to successful performance on string-pulling problems. The present study aims to investigate what strategies are used by Western scrub-jays (*Aphelocoma californica*) when confronted with various multiple-string problems. While a lot of work on the cognition behind string-pulling has focused on the genus *Corvus,* to date little is known about the abilities of other corvids in these tasks. Assessing the performance of a more distantly related genus, *Aphelocoma*, will be informative as to the distribution of physical cognition across the *Corvidae.*

**Fig. 1:**
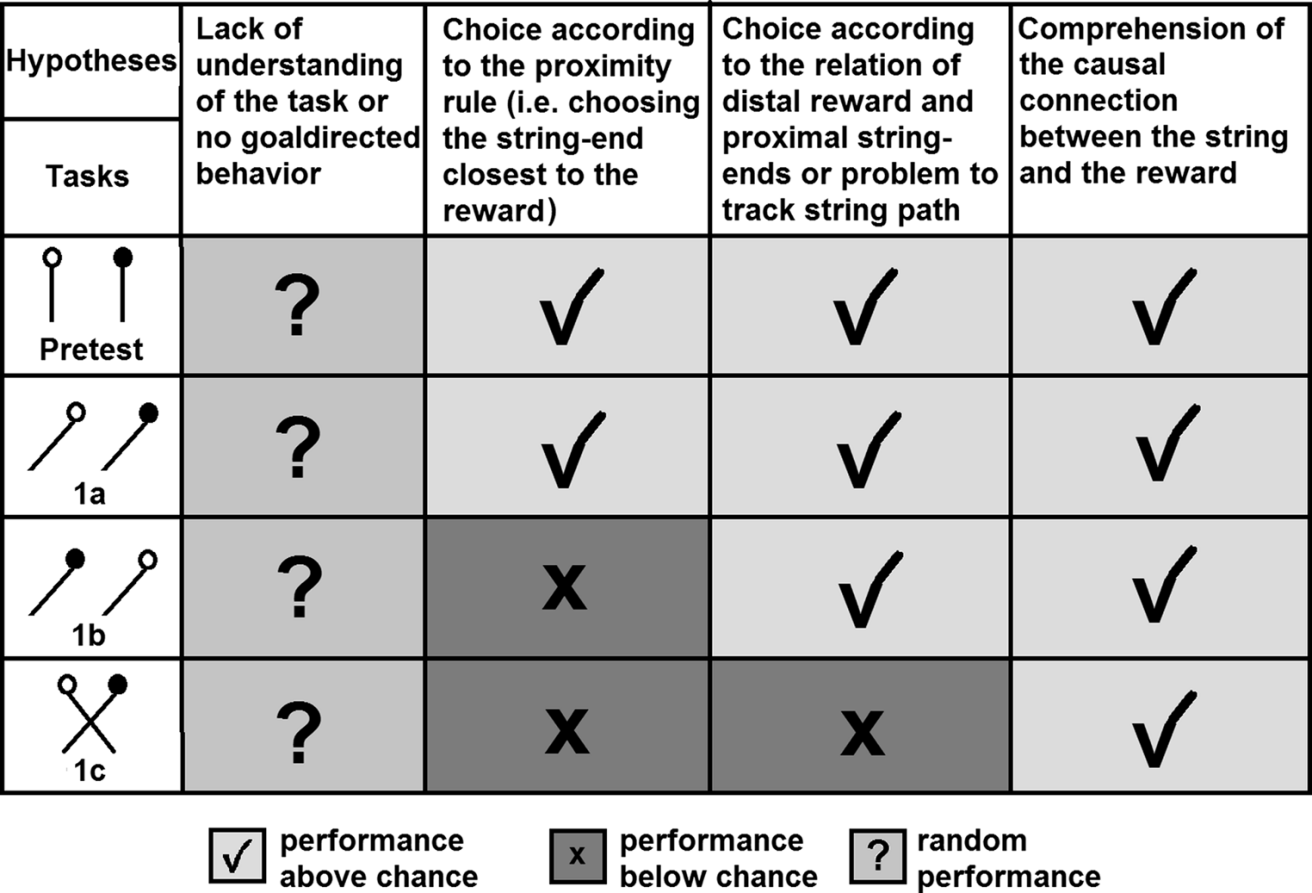
Potential response rules of the scrub-jays and their predicted outcome for the tasks of Experiment 1

In order to assess the performance of Western scrub-jays on string-pulling tasks, we conducted two experiments. In Experiment 1 the birds were tested in three patterned string problems. In these problems the subject has to choose between two or more strings arranged in different patterns, of which only one is connected to the reward. In the “Pretest”, the strings lay parallel with only one string baited. This allowed assessment as to whether scrub-jays can discriminate between rewarded and unrewarded strings and whether their behaviour towards the string is goal-directed. Two slanted-string tasks were designed to investigate the rules by which scrub-jays chose which string to pull. In the easier version of this problem (Experiment 1a, see Fig. 4), the baited string is on the outside and therefore the reward is closest to the string-end connected to it. In the more difficult version of this task (Experiment 1b, see Fig. 4) the baited string is on the inside and therefore the reward is closest to the unrewarded string-end. These tasks assessed whether the scrub-jays would follow the path of the string and pull only the baited string, or whether they would choose instead the string-end closest to the reward (“proximity rule”). If the birds chose according to proximity this would point towards a lack of understanding of the underlying physical principles. If they, on the other hand, succeeded in both slanted tasks, this would suggest some comprehension of the causal connection between the string and the reward. Additionally, the scrub-jays were tested on a crossed-string problem (Experiment 1c, see Fig. 4). This task enabled us to investigate whether the birds attended to the continuity of the strings or merely the relation of distant food and proximate tool, i.e. if the birds succeeded in Experiments 1a and 1b but failed 1c this would indicate that they do not choose according to the proximity rule, but consider the relative position of food and string-end; for example, pulling the left string if the food is on the left. A summary of the potential response rules of the scrub-jays and their predicted outcomes in terms of performance on Experiment 1 is shown in Fig. 1.

**Fig. 2:**
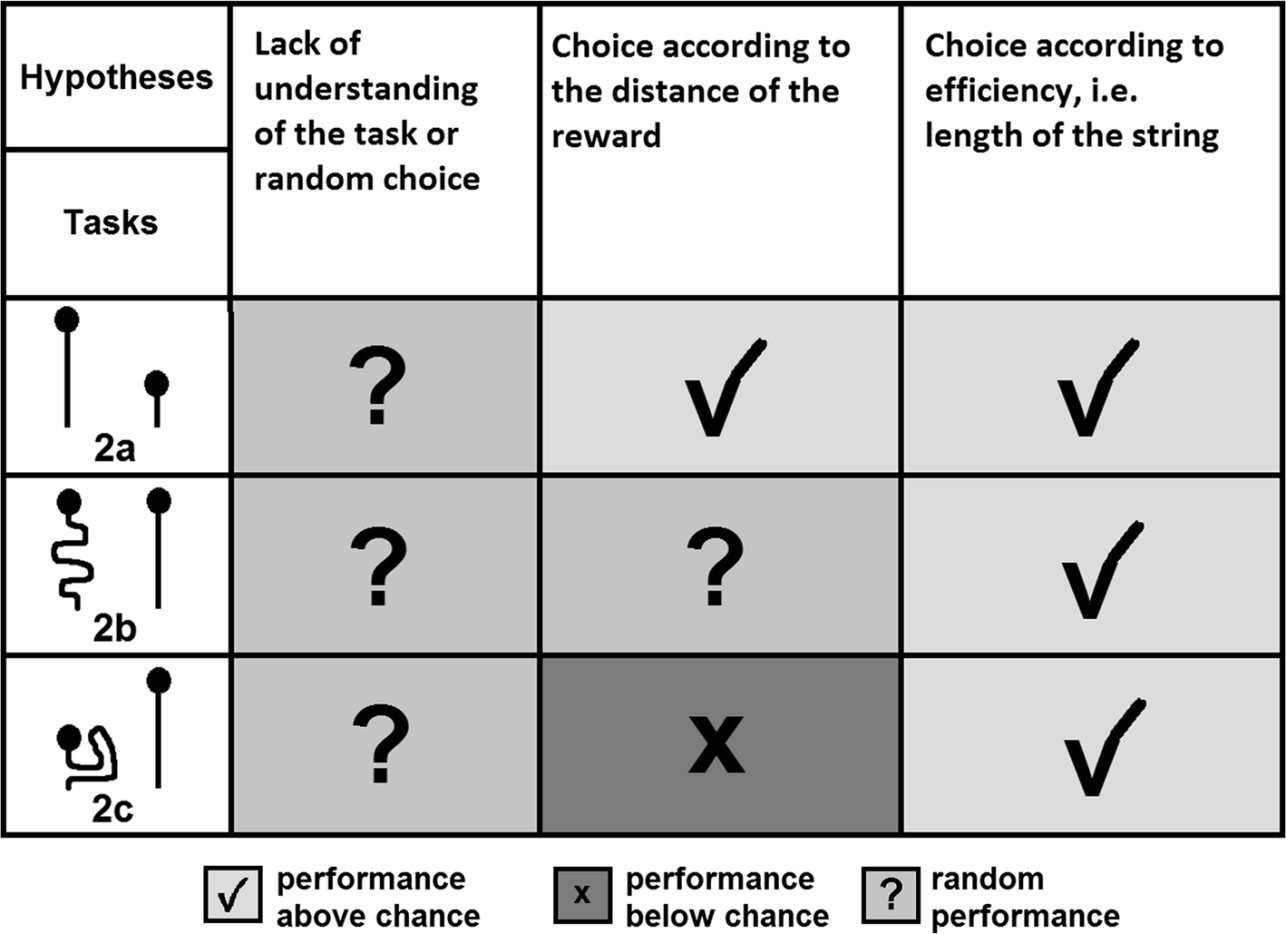
Hypotheses for problem-solving mechanisms and the predicted outcome for the tasks of Experiment 2

In Experiment 2 the scrub-jays were required to distinguish between two strings of different lengths, both of which were rewarded. In the first task (Experiment 2a, see Fig. 4) both strings were arranged straight and parallel, and thus, the reward attached to the longer string was further away from the bird. In the second task (Experiment 2b, see Fig. 4) the reward attached to the longer string was presented at the same distance from the bird as that attached to the short string (i.e. the longer string was positioned with a great deal of slack). Finally, in the third task (Experiment 2c, see Fig. 4), the reward attached to the longer string was closer to the bird than the reward attached to the short string. By manipulating the spatial arrangement of the rewards it was possible to distinguish between cues used by the birds to solve the task. If the birds understood the underlying physical principles they should prefer the shorter and thus more efficient string in all three tasks. If the birds were guided by the reward distance irrespective of the string characteristics they should consistently choose the string-end corresponding to the food item closest to them. A summary of the hypotheses and predicted outcomes of Experiment 2 is shown in Fig. 2.

Moreover, this set-up also allowed for studying the effect of visual feedback on the birds. Taylor et al. (2010, 2012) put forward the perceptual feedback hypothesis, suggesting that a steady approach of a reward item when pulling the correct string would reinforce this activity whereas the lack of reward movement would reduce the birds’ pulling motivation strongly. Since the long string had to take up slack in Experiments 2b and 2c before the reward approached, it was possible to check for the effect of feedback on scrub-jays as well. An absence of reward movement could lead the bird to change the string and switch to the other option.

## General methods

### Subjects

Eleven Western scrub-jays (*Aphelocoma californica*), six males and five females, started with the experiments, but due to reasons explained in the section “General methods”, Training only five birds finished all the tasks. The hand-raised birds were adults aged between 7 and 16 years and kept at the University of Cambridge’s Sub-department of Animal Behaviour. For individual recognition the birds were banded with coloured leg rings and identified by numbers indicating their age (for a detailed overview, see supplementary material). They were pair-housed in indoor cages (2 m wide × 1 m high × 1 m deep) in climate-controlled rooms (temperature 21 °C) and maintained on a mixed diet (cat kibble, eggs, nuts, vegetables, seeds and fruit). Maintenance diet was removed 2 h preceding testing and during experiments, but birds had access to water ad libitum. Lighting was provided according to a 12 h light/12 h dark cycle. Maintenance of the animals followed the University of Cambridge and UK Home Office guidelines. All subjects had been used for various studies before, but had no experience of string-pulling.

### Experimental design

**Fig. 3:**
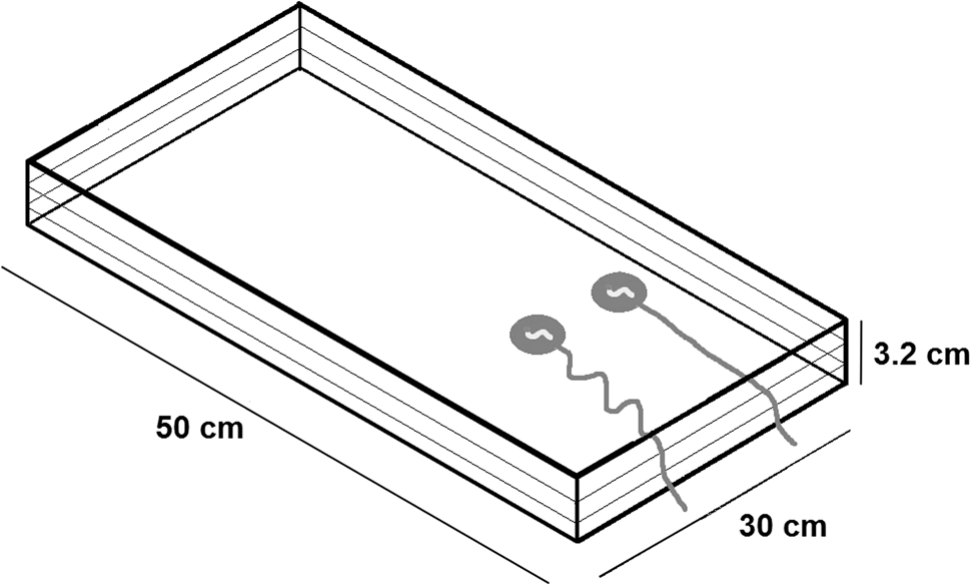
Apparatus, example set-up for Experiment 2B

All experiments were conducted using a transparent Perspex box (30 × 3.2 × 50 cm, see Fig. 3) consisting of three transparent Perspex plates, of which the top two were removable. This created a two-level set-up allowing for experiments with visually crossed strings that did not touch each other. The rewards were wax moth larvae, “wax worms” (except for Bird 229 who preferred pumpkin seeds) presented in white lids of milk cartons, which were connected to white strings (0.2 mm in diameter) of 14 or 24 cm length. The lids had a diameter of 3.2 cm and were 0.8 cm high. Thus, the subjects were able to see the reward from the top whereas it was not visible from the side. Therefore, the birds were required to inspect the set-up from above in order to make a choice before they pulled a string.

**Fig. 4:**
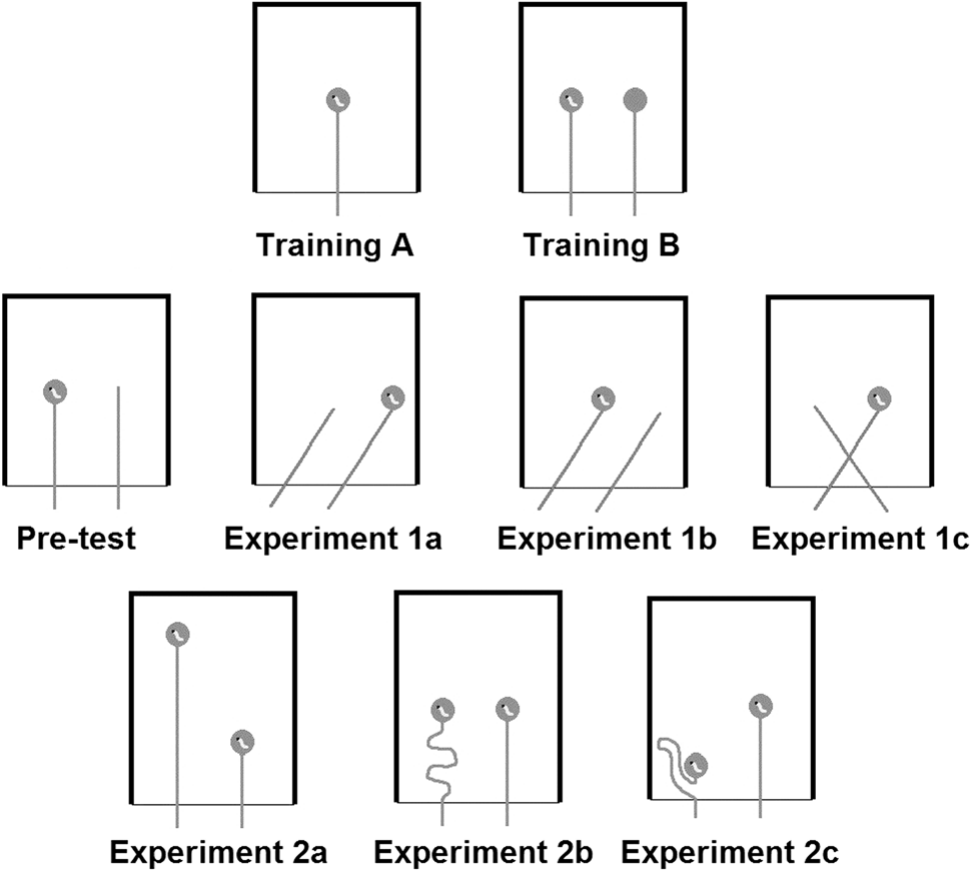
String arrangement of Training, Experiment 1 and Experiment 2

The strings were arranged in different spatial relations according to the task (see Fig. 4). In the Training Phase the birds were firstly presented with a single string (14 cm) perpendicular to the edge of the perspex box (Training A) and secondly with two parallel strings (14 cm) where only one of them was baited (Training B). In Experiment 1 the strings (14 cm) were either arranged parallel, slanted or crossed, and in Experiment 2 the distance of the reward attached to the longer string (24 cm) was changed in relation to the reward attached to the shorter string (14 cm). The distance between the string-ends was about 10 cm in all tasks with the amount of string protruding from the box approximately 2–3 cm.

### Testing procedure

The training and experiments were conducted between October 2013 and June 2014 (see supplementary material for the precise dates of testing each birds). All subjects were tested individually on horizontal two-string discrimination tasks. After 2 h of food deprivation the birds were isolated into a 2 m × 1 m × 1 m testing cage such that they had acoustic, but not visual, access to their partner.

In the testing cage the birds were presented with the Perspex box containing strings arranged according to the specific experiment. The rewarded side was counterbalanced such that no one side was baited more than twice in succession. To avoid giving any cues during the process of arranging the string for a new trial, both strings were always manipulated at the beginning of each trial. During the experimental session trials were conducted in immediate succession.

An experimental session lasted a maximum of 1 h per day. If the subject did not approach the apparatus for more than 10 min, the session ended earlier. After both of the pair-housed individuals finished the session the scrub-jays were reunited. Deprivation ended after a maximum of 4 h, isolation from their mate after a maximum of 2 h.

### Analysis

All trials were recorded using Geovision GV-1480 CCTV © 2006. Three coders independently coded a random selection of 10 % of the videos for each experiment. For inter-observer reliability the unweighted Cohen’s kappa for nominal data was calculated (Cohen 1960). It was never below *k* = 0.98, which means almost perfect inter-observer reliability. As all tasks were discrimination tasks with two different possibilities a binomial test was applied to compare the birds’ performance to chance. The number of correct responses out of the total number of trials was tested against a chance level of 50 %. Due to the relatively small numbers of birds, nonparametric statistics were used throughout the analysis. For the comparison of the performances of the first and second half of trials Fisher’s exact test was used and the Bonferroni correction was applied in order to correct for the effects of multiple testing. To analyse the switching behaviour in Experiment 2, the Kruskal–Wallis test was applied. Data analysis was conducted using the software program R (R Project for Statistical Computing, http://www.r-project.org/). Significance was set at *α* = 0.05. All statistical tests were two-tailed.

#### Ethical approval

All applicable international, national and institutional guidelines for the care and use of animals were followed. Under UK law, no specific approval was required for this noninvasive study. This article does not contain any studies with human participants.

## Training

### Methods

#### Procedure

Initially the birds were habituated to the Perspex box by repeatedly eating wax worms from it. Here strings were present but were not necessary to retrieve the food. After the birds approached the box reliably, the string-pulling training began.

The first Training Phase (Training A) was conducted in three steps. In the beginning the reward was located at the very edge of the apparatus and thus could be directly obtained without pulling string. In the second stage the reward was placed approximately 3 cm inside the apparatus leaving a large length of string protruding, thus necessitating the use of the string but only requiring a very small pull. In the third stage the reward was placed approximately 10 cm within the Perspex box, such that only a short piece of string protruding from the box. After a bird was performed each of the three stages to criterion (five successful trials in a row), the second Training Phase started. This training procedure was not conducted with subject 210, because this bird already successfully solved stage three during the familiarization process and repeated this behaviour reliably in all subsequent trials.

In the second Training Phase (Training B) two strings were simultaneously presented to the subjects. The strings were the same as the ones used in the first Training Phase. One of the lids was baited with a waxworm, whereas the other lid remained empty. The strings were arranged parallel to one another and perpendicular to the opening of the box (see Fig. 3). The baited side was alternated randomly across trials, but no side was baited more than twice in a row.

## Results and discussion

Ten of the eleven tested birds passed the first Training Phase. Bird 202 was unwilling to approach the apparatus and therefore was not used for further experiments. In the second Training Phase, only one of the remaining ten scrub-jays performed significantly above chance. While Bird 229 had a success rate of 68 % (*P* = 0.002 in a binomial test with a probability of success of 50 %), the average probability of success lay at 51 % with none of the other results significantly different from chance level (although Bird 222 had a success rate of 71 %, this result was not statistically significant because of a smaller number of trials before reaching the criterion of five successful trials in a row).

During the course of the training and experiments some birds developed a strong side bias, started breeding or did not perform a sufficient number of trials per day and were therefore excluded from further testing. As a result only five birds (three males and two females) were taken forward into the main experiments. These birds were not chosen according to their training performance but because they remained testable and did not develop any of the behaviours depicted above, which made exclusion necessary.

Given the problems in Training Phase two, where most of the birds did not solve the basic task of perpendicular parallel strings, it was necessary to redesign the task so as to make the location of the reward more obvious to the birds. This was achieved by removing the lid of the unrewarded string, therefore contrasting a bare string against one with a rewarded lid. Since the birds were already capable of pulling one string out of the Perspex box, no further training was conducted with the lid/no-lid set-up, but the parallel arrangement of two strings as used in Training Phase two was used for testing. As it can be seen in the following section, the new design facilitated the birds to better discriminate the strings and thus solve some of the tasks.

### Experiment 1

Experiment 1 consisted of three of the most common patterned string problems, namely parallel strings, slanted strings and crossed strings, comparable to tasks I, II and VI of Harlow and Settlage’s patterned string design (1934), see Fig. 4. There were two possibilities for the slanted-string task. Either the reward was closest to the connected string-end (Experiment 1a) or it was closest to the unconnected string-end (Experiment 1b).

### Methods

#### Subjects

Five birds (203, 207, 210, 220 and 229) took part in this experiment.

#### Procedure

Each individual received a total of 50 trials per task, i.e. a total of 200 trials, where the birds had to choose between two strings of 14 cm length. Each trial ended when a string (either rewarded or unrewarded) was removed from the box, which was usually achieved with a single pull. The position of the reward was varied pseudo-randomly where no side was rewarded more than twice in a row.

The first twenty trials of each task were conducted according to their expected difficulty following the suggestions of Wasserman et al. (2013), i.e. firstly the parallel-string task (Pretest), secondly the slanted-string tasks (Experiments 1a and 1b) and thirdly the crossed-string task (Experiment 1c). The remaining 30 trials of each task were conducted in a randomly interleaved sequence. After a maximum of 10 trials of the same experimental phase another task was presented. The sequence of tasks was chosen at random and varied between the birds. This was done to prevent the formation of response habits. Squirrel monkeys, budgerigars and rock squirrels succeed in patterned string problems when given many repetitions of the same condition but fail to perform above chance in an intermixed design, suggesting that they develop task-specific biases (Cha and King 1969, but see Harris and Meyer 1971 for contrasting results; Dücker and Rensch 1977; King and Witt 1966). In order to avoid this type of learning and to test for causal understanding the tasks were presented randomly.

To avoid the movement of the strings interfering with one another in Experiment 1c, the two levels of the Perspex box were used, containing one string each. Viewed from above the strings crossed, but in fact they did not touch, thus each string could be pulled without moving the other string. Both the side which was rewarded and the level of the reward (whether in the top or the bottom level of the Perspex box) were varied pseudo-randomly without repeating the same pattern more than twice.

For the analysis only the initial choice was considered, since it took only one pull in almost all cases to retrieve the reward.

## Results and discussion

The Pretest was solved by four out of the five birds successfully (*P* < 0.05) with birds 210, 220 and 229 performing highly significantly above chance level (*P* < 0.001). Experiment 1a, where the reward was closest to the end of the rewarded string, was successfully performed by all five birds (*P* < 0.001). In the more difficult version (Experiment 1b), in which the reward was opposite the end of the unrewarded string, four out of the five birds chose randomly. Only Bird 220 chose the rewarded string significantly more often than the bare string. No bird solved Experiment 1c successfully. The numbers of pulls of the rewarded string are shown in Table 1.

**Table 1:** Number of correct choices in Experiment 1 out of 50 trials

	203	207	201	220	229
Pretest	29	34*	41***	38***	41***
Experiment 1a	47***	44***	40***	39***	47***
Experiment 1b	26	31	21	33*	21
Experiment 1c	29	21	26	24	24

Significant results (according to a two-tailed binomial test with chance level at 50 %) are bold and marked with * *α* < 0.05), ** (*α* < 0.01) or *** (*α* < 0.001)

Experiment 1 showed that scrub-jays were capable of solving some patterned string tasks while failing others. The pattern of results appears to be relatively clear: all five birds solved Experiment 1a, whereas only one bird solved Experiment 1b and no bird solved Experiment 1c. In other words, birds were generally only successful when the closest string-end to the reward was the one that was also connected. This might be taken as evidence that the subjects chose according to the proximity rule (see Fig. 1) as opposed to understanding the causal connection between the string and the reward. While this is not the most cognitively sophisticated approach to solving the problem, it makes sense from an ecological perspective. In nature it is generally the case that the means to achieve food are physically close to the food itself.

It might seem surprising that Bird 203 did not perform significantly above chance in the Pretest despite solving Experiment 1a. However, other studies have also found that some birds were successful in more complicated tasks although they failed easier ones. For instance, Bagotskaya et al. (2012) tested a hooded crow, which failed a single string task but successfully coped with multiple-string problems. In a follow-up experiment, which was conducted after all experiments of this study were completed, Bird 203 proved that he was also able to solve the Pretest. One possibility therefore is that increased experience with string-pulling could have resulted in improved performance. However, given that in the initial 50 trials he performed better in the first half of the experiment than in the second half (7 incorrect choices in the first 25 trials, 14 incorrect choices in the second 24 trials), this explanation may be simplistic.

The outcome of Experiment 1c revealed an additional aspect of the mechanisms involved in solving patterned string tasks. Since this was the only two-level task (both the bottom and the top level of the apparatus were used), a new factor was added to the experiment. Not only did the bird have to choose between left and right, but also between top and bottom. The significant preference for the top level shows the influence of habituation in these tasks. Since the birds had previously only experienced pulling strings from the top level, it is not surprising that they stuck to this scheme. Nevertheless, the result provides evidence that the cognitive mechanisms of scrub-jays involved in string-pulling may not be as complex as those of ravens (e.g. Heinrich and Bugnyar 2005), which were able to pull up a piece of meat attached to a string even when the string was diverted and thus had to be pulled down to make the meat go up.

### Experiment 2

In Experiment 2 the birds were presented with two strings of different lengths. The distance between the reward attached to those strings and the subjects varied between the three tasks (see Fig. 4). This design was chosen in order to determine whether string length, as a proxy for pulling efficiency or reward distance, is crucial for the birds’ choice of which string to pull.

### Methods

#### Subjects

The same birds as described in Experiment 1 were tested.

#### Procedure

Each individual received a total of 50 trials per task. Within each task the sides for the long and short string were varied pseudo-randomly. Each of the three tasks used the same two strings, a short string (14 cm) and a long string (24 cm). Since the shorter string was also used for the Training Phase and Experiment 1, increased familiarity to this string might have resulted in a preference. To address this problem, subjects were given three trials before being introduced to each new task where only the long string was presented, arranged according to the pattern of the task (i.e. straight, slack or with the lid close to the edge of the box) in order to familiarize them with the long string.

In Experiment 2a both strings lay straight, which led to a distance of the reward on the long side of 20 cm and a distance of the reward on the short side of 10 cm (see Fig. 3). In Experiment 2b the rewards were both placed at a distance of 10 cm from the edge of the box and the excess of the long string was slack. In Experiment 2c the reward attached to the longer string was placed about 3 cm from the edge of the box and the excess of the long string was slack. The short string was arranged in a straight line in all three tasks. After presenting the first 20 trials of Experiment 2a, then 2b and then 2c the sequence of testing was randomly interleaved with the same task no more than five times in a row.

For the analysis of Experiment 2, two variables, namely the birds’ initial and their final choice, were considered. It was necessary to consider both options, since the retrieval of the longer string required several pulls (usually 3–4), which allowed the bird to switch strings after an initial pull. This made it possible to analyse the birds’ switching behaviour, which is informative with regard to the perceptual feedback hypothesis put forward by Taylor et al. (2010, 2012). Pulling the correct string leads to a steady approach of the reward and thus reinforces this activity. This theory is currently strongly debated, because it would mean that string-pulling tasks could be solved purely by associative learning without any causal understanding (Jacobs and Osvath 2015). However, there is evidence that some mammals will continue pulling slack strings without any initial perceptual feedback, for example dogs and wolves (Frank and Frank 1985), or even continue pulling when the reward moves away, for example gibbons (Beck 1967) or baboons (Bolwig 1963). In Experiments 2b and 2c the arrangement of the longer string created a situation similar to studies with slack strings, where multiple pulls are required before the reward starts to move.

## Results and discussion

**Fig. 5:**
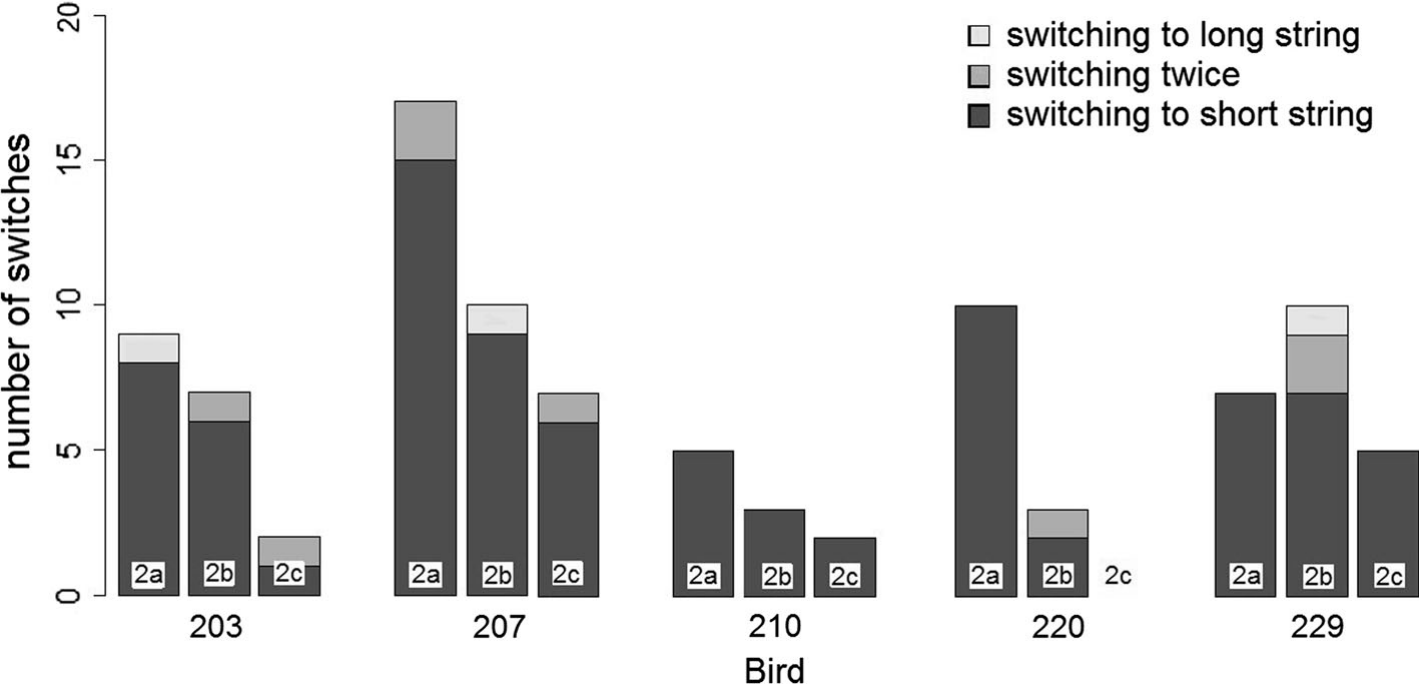
Number of switches of strings in Experiment 2 by bird and direction of the switch. “Switching twice” includes both long-short-long and short-long-short switches

In all three tasks, there was no significant preference for either string for the initial choices (see Table 2). The only exceptions were a significant preference of Bird 220 for the short string in Experiment 2b (*P* < 0.05) and a significant preference of Bird 229 for the long string in Experiment 2c (*P* < 0.05). In terms of the birds’ final choices, all birds preferred the short string significantly in Experiments 2a and 2b, whereas no bird showed a significant preference for either the short or long string in Experiment 2c (see Table 3). Analysing the switching behaviour showed a clear preference for the switch from the long to the short string. While the scrub-jays switched 86 times in this direction, the switch in the other direction occurred only three times. The birds switched twice on eight trials. Apart from Bird 229, which switched most often in Experiment 2c, the number of switches decreased from task 2a–2b–2c (see Fig. 5). Caution is required when interpreting these results. Since the short string was usually retrieved with only one pull, the birds had little opportunity to switch to the longer string. In order to correct for this bias we not only analysed the total number of pulls of each string type but also the proportion of switches which occurred after an initial long-string pull (i.e. from the long to the short string) within the different tasks (see Table 4). This analysis shows that the proportion of switches was always much higher in Experiments 2a and 2b than in Experiment 2c (Kruskal–Wallis, Chi-squared = 8.72, *df* = 2, *P* = 0.01277).

**Table 2:** Number of initial choices of the short string in Experiment 3 (out of 50 trials)

	203	207	201	220	229
Experiment 2a	26	26	32	31	30
Experiment 2b	28	31	31	33*	30
Experiment 2c	19	18	18	29	17*

Significant results (according to a two-tailed binomial test with chance level at 50 %) are bold and marked with * (*α* < 0.05), ** (*α* < 0.01) or *** (*α* < 0.001)

**Table 3:** Number of final choices of the short string in Experiment 3 (out of 50 trials)

	203	207	201	220	229
Experiment 2a	33*	41***	37***	41***	37***
Experiment 2b	34*	39***	34*	35**	36**
Experiment 2c	20	24	20	29	22

Significant results (according to a two-tailed binomial test with chance level at 50 %) are bold and marked with * (*α* < 0.05), ** (*α* < 0.01) or *** (*α* < 0.001)

**Table 4:** Proportion of long-string pulls followed by a switch

	203	207	201	220	229
Experiment 2a	0.33	0.63	0.28	0.53	0.35
Experiment 2b	0.27	0.47	0.16	0.12	0.35
Experiment 2c	0.03	0.19	0.002	0	0.15

The results of Experiment 2 indicate that two different factors influenced the scrub-jays’ pulling behaviour, namely the absolute reward distance and the movement of the reward. Although almost all birds chose the strings at random initially, they ultimately retrieved the reward attached to the short string significantly more often than the one attached to the long string in Experiments 2a and 2b, which indicates that they were sensitive to the reward’s movement and adapted their choice by switching the string when the reward was not approaching due to their pulls. However, in Experiment 2c the proportion of switches to the short string was much lower (see Table 4). This suggests that the birds were strongly motivated to achieve the reward closest to them irrespective of the effort necessary to do so. This finding is consistent with many other studies that also reported that the distance of food has an effect on the choice of both mammals and birds and can be more influential than the functionality of the tool. For instance, capuchin monkeys showed a preference for the closer food item in a hook task even if they were not able to retrieve the reward because the hooks were nonfunctional (Fujita et al. 2003). Pigeons also prefer shorter strings over longer strings in virtual string-pulling tasks, indicating that they are also influenced by the distance of the reward as well as the effort and time necessary to procure the reward (Wasserman et al. 2013). However, it is important to point out that the distance of the reward was confounded with pulling effort in the pigeon study. It might seem surprising that although the scrub-jays appeared sensitive to the reward distance, they were not initially successful in Experiment 2a, but only after switching. This might have been caused by the relatively large distance of both rewards in this task. While very close food items seemed to be highly motivating for the birds and strongly influenced their initial choices (e.g. “close” vs. “not close”), when both strings were 10 cm or more this might not have facilitated easy discrimination between two different distances (e.g. “not close” vs. “not close”).

The fact that the scrub-jays often switched string during a task indicates that they also reacted to the movement of the reward. This suggests that the birds did not form a mental representation of the task in advance: if this had been the case, we would expect them to choose the shorter string in the first instance rather than switching after observing the consequence of their pulling behaviour. A similar tendency was also reported for pigeons (Brzykcy et al. 2014) which usually corrected themselves during virtual string-pulling tasks after seeing the effect of their first action and thus had a higher percentage of correct final choices than first choices.

However, the switching does not completely fit Taylor’s perceptual feedback hypothesis. Most of the switches occurred in Experiment 2a, where both strings were arranged in a straight line and where therefore any pull caused the movement of the reward. In Experiments 2b and especially 2c, where the longer string was slack and produced little movement on the initial pulls, switching occurred more rarely (see Table 4). If the birds purely relied on the visual feedback of the approaching reward, one would expect fewer switches in Experiment 2a than 2b and 2c. It is possible, however, that instead of fully inspecting the string-reward arrangement ahead of time, the birds used the movement of the reward after an initial pull to ascertain which string was attached to which reward, and then switched their choice if it was revealed that they were pulling the string attached to the more distant reward.

The observed switching pattern could be explained by an additional factor. Due to previous experience with other string-pulling tasks the birds might have been used to a success after a single pull and thus switched when this success did not immediately occur. The reduced number of switches in Experiment 2c might point towards a strong influence of the distance of the reward. The birds might have strongly been attracted by the closeness of the reward in task 2c and therefore continued pulling the long string even if the reward did not initially move. Thus, a combination of reward distance, its movement and previous experience seem to be the best explanation for the observed results.

## General discussion

The objective of this study was to test physical cognition in Western scrub-jays by presenting them with a selection of horizontal string-pulling tasks across two experiments. This is the first investigation of physical cognition in scrub-jays despite extensive research on their impressive cognitive abilities in other contexts. Experiment 1 assessed whether or not scrub-jays took account of connectivity, while Experiment 2 examined the jays’ ability to choose the most efficient string.

Experiment 1 did not provide evidence that the jays understood the functionality of a string. Instead, they appeared to rely on a “proximity rule”, pulling the string-end nearest to the reward. This preference for proximity was reflected in the results of Experiment 2, where the jays’ switching behaviour was influenced by the proximity of the reward. A reliance of primates on the proximity of the reward has already been shown by Harlow and Settlage (1934) and was reported to varying degrees in many studies for other animals as well, for instance dogs (Osthaus et al. 2004), squirrel monkeys (Cha and King 1969), and even apes and crows (Albiach-Serrano et al. 2012). It should be noted, however, that the scrub-jays did not faithfully follow this proximity rule. Had they done so, they would have been expected to perform below chance on Experiment 1b and Experiment 1c (see Fig. 1). However, the results of these tasks did not significantly differ from a random choice. This could be due to short-term learning of the birds, since after a few unsuccessful trials they might have changed their strategy. The number of trials per session was not sufficient to analyse this hypothesis statistically.

In Experiment 2 the scrub-jays did not show a preference for the shorter and therefore more efficient of two strings in their initial choices, but did so in their final choice in two of the three tasks, because they showed a high proportion of switches to the short string when their initial choice was the long string. When the arrangement of the strings provided two contradictory cues, namely when the reward attached to the longer and thus inefficient string lay closer to them, the birds did not react with a switch to the short string as often as in the other tasks, but stuck to their initial choice, potentially motivated by the proximity of the reward.

Although the jays appeared to choose a string at random initially, they adapted their choice after observing the results of their behaviour in two of three tasks. This pattern of behaviour fits the hypothesis of Taylor and colleagues (Taylor et al. 2010, 2012), who claimed that the good performance of many species of corvid in the string-pulling tasks, even that of ravens, can be explained by a “perceptual-motor feedback loop” rather than a comprehension of the means-end relation of string and reward. If the corvids comprehended the physical rules of string-pulling, then they should be able to perform successfully even if the visual feedback is limited, and in cases where (as in this study) the correct choice should be visually obvious without having to first pull a string. This perceptual-motor feedback account, which essentially posits the approach of food as a positive reinforcer driving associative learning, has also been suggested as the underlying learning mechanism in other physical cognition paradigms such as the aesops fable task (Cheke et al. 2011, 2012) and could theoretically underpin much tool-using behaviour in animals.

Scrub-jays appear to use a combination of simple response rules when confronted with string-pulling problems. A preference for rewards that are physically closer makes sense in a foraging context; usually the closer rewards are easier to obtain. However, the jays did not stubbornly rely on fixed rules but showed flexibility in their responses, which became evident in two ways. Firstly, they did not perform significantly below chance, which would have been expected in Experiment 1b or 1c if they relied completely on the proximity rule. Secondly, they switched between strings in Experiment 2 in about 10 % of all trials, but the vast majority of these switches were from the less efficient to the more efficient string, and only when the reward attached to the string was not in their direct proximity. The fact that the birds attended to such perceptual cues and adapted their strategy in the event of a failure could be a precursor to physical problem-solving, providing the basis for the development of causal understanding. Indeed, Schmidt and Cook (2006) argued that attending to the relevant perceptual cues is a crucial initial step towards the development of an understanding of the means-end relations. It is possible that had we used longer strings (that is, strings that required more than one pull) in Experiment 1 we would have also observed this switching behaviour and the birds might have retrieved the reward on a higher proportion of trials.

This study represents the first attempt to investigate physical cognition in scrub-jays. By explicitly testing potential rules for solving string-pulling tasks we can not only compare their results with studies of other species, but also investigate the learning rules by which scrub-jays approach such tasks. The results suggest that these birds may not understand the causal mechanisms underlying string-pulling tasks. Instead, they appear to use a range of simpler response rules focusing on the spatial arrangement of the reward. Generally, the results align nicely with findings of other string-pulling studies, suggesting that the scrub-jays perform similarly to most of the other corvids and parrots that have been tested (see Jacobs and Osvath 2015 for an overview of over 200 string-pulling studies). A complete comprehension of the functionality of strings is rare in the animal kingdom and has so far been suggested only for ravens (Heinrich 1995) and some primates (e.g. Mayer et al. 2014). Strategies like the proximity rule, however, may be relatively common across species, e.g. corvids (Bagotskaya et al. 2012; Taylor et al. 2010), squirrel monkeys (Cha and King 1969), common marmosets (Gagne et al. 2012), apes (Köhler 1927), rhesus monkeys (Mason et al. 1956) and parrots (Schuck-Paim et al. 2008). The results of this study seem to suggest that the jays’ remarkable achievements in caching studies may not extend to string-pulling tasks as an example for physical understanding. However, since New Caledonian crows, which are well known for their good physical cognition, struggle with some of the string-pulling tasks we presented, further investigations of the jays’ physical problem-solving capacities with different paradigms are necessary to confirm these findings. For instance, it would be interesting to test the scrub-jays on other benchmark tests of physical cognition, like the two-trap trap-tube test (Seed et al. 2006; Taylor et al. 2009a, b) or the Aesop’s fable water task (Bird and Emery 2009b; Cheke et al. 2011, 2012; Jelbert et al. 2014). By investigating their performance on a broad range of cognitive tasks, assessing different types of intelligence, we may develop a more nuanced understanding of the generality of intelligence in animals.

## Electronic supplementary material

Below is the link to the electronic supplementary material.

Supplementary material 1 (DOCX 13 kb)

Supplementary material 2 (PDF 322 kb)

## References

[CR1] Albiach-Serrano A, Bugnyar T, Call J (2012). Apes (*Gorilla gorilla, Pan paniscus, P. troglodytes, Pongo abelii*) versus corvids (*Corvus corax, C. corone*) in a support task: the effect of pattern and functionality. Comp Psychol.

[CR2] Bagotskaya MS, Smirnova AA, Zorina ZA (2012). Corvidae are able to understand the logical structure in string-pulling tasks. Neurosci Behav Physiol.

[CR3] Beck BB (1967). A study of problem solving by gibbons. Behaviour.

[CR4] Bird CD, Emery NJ (2009). Insightful problem solving and creative tool modification by captive nontool-using rooks. Proc Natl Acad Sci USA.

[CR5] Bird CD, Emery NJ (2009). Rooks use stones to raise the water level to reach a floating worm. Curr Biol.

[CR6] Bolwig N (1963). Observations on the mental and manipulative abilities of a captive baboon (*Papio Doguera*). Behaviour.

[CR7] Brzykcy SJ, Wasserman EA, Nagasaka Y, Perez-Acevedo S (2014). Validating the virtual string task with the gap test. Anim Cognit.

[CR8] Cha JH, King JE (1969). The learning of patterned strings problems by squirrel monkeys. Anim Behav.

[CR9] Chappell J, Kacelnik A (2002). Tool selectivity in a non-primate, the new Caledonian crow (*Corvus moneduloides*). Anim Cognit.

[CR10] Chappell J, Kacelnik A (2004). Selection of tool diameter by new Caledonian crows *Corvus moneduloides*. Anim Cognit.

[CR11] Cheke LG, Bird CD, Clayton NS (2011). Tool-use and instrumental learning in the Eurasian jay (*Garrulus glandarius*). Anim Cognit.

[CR12] Cheke LG, Loissel E, Clayton NS (2012). How do children solve Aesop’s fable?. PLoS One.

[CR13] Cohen J (1960). A coefficient of agreement for nominal scales. Educ Psychol Meas.

[CR14] Del Hoyo J (1992). Handbook of the birds of the world.

[CR15] Dücker G, Rensch B (1977). The solution of patterned string problems by birds. Behaviour.

[CR16] Emery NJ (2004) Are corvids “feathered apes”? Cognitive evolution in crows, jays, rooks and jackdaws. In: Comparative analysis of minds. Keio University Press, Tokyo, pp 181–213

[CR17] Emery NJ, Clayton NS (2004). The mentality of crows: convergent evolution of intelligence in corvids and apes. Science.

[CR18] Frank H, Frank MG (1985). Comparative manipulation-test performance in ten-week-old wolves (*Canis lupus*) and Alaskan malamutes (*Canis familiaris*): a Piagetian interpretation. J Comp Psychol.

[CR19] Fujita K, Kuroshima H, Asai S (2003). How do tufted capuchin monkeys (*Cebus apella*) understand causality involved in tool use?. J Exp Psychol Anim B.

[CR20] Gagne M, Levesque K, Nutile L, Locurto C (2012). Performance on patterned string problems by common marmosets (*Callithrix jacchus*). Anim Cognit.

[CR21] Harlow HF, Settlage PH (1934). Comparative behavior of primates. VII. Capacity of monkeys to solve patterned string tests. J Comp Psychol.

[CR22] Harris DG, Meyer ME (1971). Performance of squirrel monkeys on systematic or random presentation of patterned string problems. Psychon Sci.

[CR23] Heinrich B (1995). An experimental investigation of insight in common ravens (*Corvus corax*). Auk.

[CR24] Heinrich B, Bugnyar T (2005). Testing problem solving in ravens: string-pulling to reach food. Ethology.

[CR25] Hunt GR (2014). New Caledonian crows’ (*Corvus moneduloides*) pandanus tool designs: diversification or independent invention?. Wilson J Ornithol.

[CR26] Jacobs IF, Osvath M (2015). The string-pulling paradigm in comparative psychology. J Comp Psychol.

[CR27] Jelbert SA, Taylor AH, Cheke LG, Clayton NS, Gray RD (2014) Using the aesop’s fable paradigm to investigate causal understanding of water displacement by new Caledonian crows. PLoS One, 9(3): e92895. doi:10.1371/journal.pone.0092895. doi:10.1676/13-085.1 10.1371/journal.pone.0092895 PMC3966847 24671252

[CR28] King JE, Witt ED (1966). The learning of patterned strings problems by rock squirrels. Psychon Sci.

[CR29] Köhler W (1927). The mentality of apes (2nd rev. ed.). (E. Winter, Trans.).

[CR30] Lefebvre L, Nicolakakis N, Boire D (2002). Tools and brains in birds. Behaviour.

[CR31] Mason WA, Blazek NC, Harlow HF (1956). Learning capacities of the infant rhesus monkey. J Comp Physiol Psychol.

[CR32] Mayer C, Call J, Aliach-Serrano A, Visalerghi E, Sabbatini G, Seed A (2014). Abstract knowledge in the broken-string problem: evidence from nonhuman primates and pre-Schoolers. PLoS One.

[CR33] Osthaus B, Lea SEG, Slater AM (2004). Dogs (*Canis lupus familiaris*) fail to show understanding of means-end connections in a string-pulling task. Anim Cognit.

[CR34] Schmidt GF, Cook RG (2006). Mind the gap: means–end discrimination by pigeons. Anim Behav.

[CR35] Schuck-Paim C, Borsari A, Ottoni EB (2008). Means to an end: neotropical parrots manage to pull strings to meet their goals. Anim Cognit.

[CR36] Seed AM, Tebbich S, Emery NJ, Clayton NS (2006). Investigating physical cognition in rooks, *Corvus frugilegus*. Curr Biol.

[CR37] Taylor AH, Hunt GR, Medina FS (2009). Do new Caledonian crows solve physical problems through causal reasoning?. Proc R Soc Lond B Biol Sci.

[CR38] Taylor A, Roberts R, Hunt G, Gray R (2009). Causal reasoning in New Caledonian crows: ruling out spatial analogies and sampling error. Commun Integr Biol.

[CR39] Taylor A, Medina FS, Holzhaider JC, Hearne LJ, Hunt GR, Gray RD (2010). An investigation into the cognition behind spontaneous string pulling in new Caledonian crows. PLoS One.

[CR40] Taylor A, Elliffe DM, Hunt GR, Emery NJ, Clayton NS, Gray RD (2011). New Caledonian crows learn the functional properties of novel tool types. PLoS One.

[CR41] Taylor A, Knaebe B, Gray RD (2012). An end to insight? New Caledonian crows can spontaneously solve problems without planning their actions. Proc R Soc Lond B Biol Sci.

[CR42] Wasserman EA, Nagasaka Y, Castro L, Brzykcy SJ (2013). Pigeons learn virtual patterned-string problems in a computerized touch screen environment. Anim Cognit.

[CR43] Weir AS, Chappell J, Kacelnik A (2002). Shaping of hooks in new Caledonian crows. Science.

[CR44] Werdenich D, Huber L (2006). A case of quick problem solving in birds: string pulling in keas, Nestor notabilis. Anim Behav.

