# Supplementary material for: Western scrub-jays (Aphelocoma californica) solve multiple-string problems by the spatial relation of string and reward
Source: Anim Cogn. 2016 Jul 28;19(6):1103–14. doi: 10.1007/s10071-016-1018-x (PMC5054065; doi:10.1007/s10071-016-1018-x)
Supplement: Supplementary file 1 — Supplementary material 1 (DOCX 13 kb) [file 10071_2016_1018_MOESM1_ESM.docx]

#### Supplementary material

Overview of the tested subjects

| Bird-ID | Gender | Ring colors | Pair-housed with | Room | Hatched in | Experiments | If not tested: reason |
| --- | --- | --- | --- | --- | --- | --- | --- |
| 31 | M | gold/red | 202 | 12 | April 1997 | Training phase | strong preferences for a side |
| 108 | M | gold/blue | 215 | 8 | April 2003 | Training phase | strong preferences for a side |
| 202 | F | purple/blue | 31 | 12 | April 2006 | Training phase | unwilling to approach apparatus |
| 203 | M | black/orange | 207 | 12 and 13 | April 2006 | All tasks |  |
| 207 | F | blue/green | 203 | 12 and 13 | April 2006 | All tasks |  |
| 210 | M | pink/green | 13 | 12 | April 2006 | All tasks |  |
| 215 | F | red/blue | 108 | 8 | April 2006 | Training phase | Insufficient number of trials per day (average: 4) |
| 220 | M | red/yellow | 229 | 8 and 13 | April 2006 | All tasks |  |
| 222 | M | orange/green | 224 | 13 | April 2006 | All tasks | started breeding (April/May 2014) |
| 224 | F | pink/yellow | 222 | 13 | April 2006 | All tasks | started breeding (April/May 2014) |
| 229 | F (?) | purple/yellow | 220 | 8 and 13 | April 2006 | All tasks |  |

Remark: Bird 13, pair-housed with 108, was not tested because of her advanced age
