# Supplementary material for: Western scrub-jays (Aphelocoma californica) solve multiple-string problems by the spatial relation of string and reward
Source: Anim Cogn. 2016 Jul 28;19(6):1103–14. doi: 10.1007/s10071-016-1018-x (PMC5054065; doi:10.1007/s10071-016-1018-x)
Supplement: Supplementary file 2 — Supplementary material 2 (PDF 322 kb) [file 10071_2016_1018_MOESM2_ESM.pdf]

Bird-ID	Ring colours	Pair-housed with	Room	Hatched in	Experiments	Gender	Period of testing
31	gold/red	202	12	April 1997	Training phase	M	11.11. - 04.12.2013
108	gold/blue	215	8	April 2003	Training phase	M	7.10. - 19.11.2013
202	purple/blue	31	12	April 2006	Training phase	F	11.11. - 22.11.2013
203	black/orange	207	12 and 13	April 2006	All tasks	M	06. - 08.11.2013,<br>11. - 06.01.2014,<br>23.04. - 14.05.2014,<br>17. - 20.06.2014
207	blue/green	203	12 and 13	April 2006	All tasks	F	06. - 08.11.2013,<br>10.04. - 14.05.2014,<br>17. - 20.06.2014
210	pink/green	13	12	April 2006	All tasks	M	11.11. - 13.12.2013,<br>08.01. - 06.02.2014
215	red/blue	108	8	April 2006	Training phase	F	07.10. - 19.11.2013
220	red/yellow	229	8 and 13	April 2006	All tasks	M	07.10 - 13.12.2013,<br>08.01. - 24.03.2014
222	orange/green	224	13	April 2006	All tasks	M	06. - 08.11.2013, 11. - 17.01.2014<br>(breeding in April/May 2014)
224	pink/yellow	222	13	April 2006	All tasks	F	06. - 08.11.2013, 11. - 17.01.2014<br>(breeding in April/May 2014)
229	purple/yellow	220	8 and 13	April 2006	All tasks	F (?)	07.10. - 09.12.2014,<br>08.01. - 24.03.2014,<br>13.05. - 11.06.2014
